# Neighborhood Opportunity and Early Life Indicators of Respiratory Health in Children Born Very Preterm

**DOI:** 10.1002/ppul.71501

**Published:** 2026-02-09

**Authors:** Ella M. Whitman, Marissa Hauptman, Lystra P. Hayden, Jonathan C. Levin

**Affiliations:** ^1^ Boston College Global Observatory on Planetary Health Chestnut Hill Massachusetts USA; ^2^ University of Vermont Larner College of Medicine Burlington Vermont USA; ^3^ Division of General Pediatrics Boston Children's Hospital Boston Massachusetts USA; ^4^ Department of Pediatrics Harvard Medical School Boston Massachusetts USA; ^5^ Region 1 New England Pediatric Environmental Health Specialty Unit Boston Massachusetts USA; ^6^ Division of Pulmonary Medicine Boston Children's Hospital Boston Massachusetts USA; ^7^ Division of Newborn Medicine Boston Children's Hospital Boston Massachusetts USA

**Keywords:** child opportunity, longitudinal outcomes, prematurity, respiratory health

## Abstract

**Background:**

Bronchopulmonary dysplasia (BPD) is the most common complication of prematurity, contributing to long term adverse respiratory outcomes that persist across the life course. However, it remains unclear how childhood opportunity impacts the underlying risk for developing BPD and post‐discharge respiratory health, both of which may impact long term outcomes.

**Methods:**

Observational cohort of 845 children born very preterm (≤32 weeks), followed for post‐prematurity respiratory disease. We derived childhood opportunity index (COI) from the census tract corresponding to each subject's address. Linear regression was used to identify the impact of COI on neonatal and childhood respiratory outcomes. In a secondary analysis, we examined differences in outcomes between races (White, non‐White), across quartiles of COI.

**Results:**

Children residing in neighborhoods with lower COI were born at a significantly smaller birth weight, earlier gestation, and spent longer duration on mechanical ventilation (MV) in the NICU. No direct association was observed between COI and longer‐term respiratory outcomes. Racial disparities in birth outcomes within COI quartiles became more pronounced at higher levels of opportunity. Longer duration of MV in the NICU was significantly associated with longer‐term outcomes including increased hospital readmissions in early life and lower FEV_1_ and FVC % predicted in childhood.

**Conclusion:**

Low COI is associated with longer duration of MV in the NICU, which itself is associated with increased healthcare utilization and reduced functional respiratory outcomes. Racial disparities in birth outcomes within similar neighborhood contexts demonstrate the need for targeted interventions to advance health equity in this population of vulnerable infants.

## 1 Introduction

Bronchopulmonary dysplasia (BPD) is the most common complication of preterm birth, affecting 10,000 to 15,000 infants in the United States each year [1]. Rates of BPD have increased in recent years despite significant advances in neonatal care, largely due to increased survival of infants born at very early gestational ages. Survival among extremely preterm infants improved by approximately 20% between 1997 and 2021, while the prevalence of chronic lung disease increased by about 10% over the same period [2]. Common risk factors for developing BPD include fetal growth restriction, adverse birth outcomes: low birth weight (BW) and early gestational age (GA), infection, and prolonged use of invasive mechanical ventilation (MV) in the neonatal intensive care unit (NICU) [3]. Children with BPD are at increased risk for lifelong respiratory morbidity and lower peak lung function [4, 5, 6, 7].

Emerging evidence has demonstrated that specific social and environmental factors influence respiratory morbidity in children with BPD, including household sources of indoor air pollution [8], residential ambient air pollution [9], number of children in the household [10], daycare attendance [11], and type of insurance coverage [12].

Beyond individual exposure analyses, composite measures of a child's social and environmental context are a powerful means to ascertain cumulative risk and better capture the complex, interacting factors that shape child development and health outcomes [13]. The Childhood Opportunity Index (COI) is a multidimensional population‐based tool that captures the quality of neighborhood resources and conditions essential for children's growth and development at the census tract level [14]. Among the unique population of infants with post‐prematurity respiratory disease, low COI is closely linked with adverse outcomes, including increased morbidity in the NICU [15], altered somatic growth trajectories [16], trends towards longer use of home oxygen therapy [17], and increased ED visits [18]. Low COI is also associated with reduced odds of participation in infant follow‐up programs, regardless of residential distance from the clinic [19].

It is likely that these socioenvironmental factors begin their impact in utero and therefore may impact the risk of developing BPD in addition to longer‐term respiratory outcomes. We conducted a retrospective analysis of an urban single‐center longitudinal registry of children with post‐prematurity respiratory disease (PPRD), evaluating the impact of COI and race on neonatal and childhood respiratory outcomes. We hypothesized that lower COI would be associated with adverse early life and longitudinal indicators for respiratory health in children born very preterm.

## 2 Methods

### 2.1 Study Population

We conducted a retrospective cohort study using data from 845 participants enrolled in the Center for Healthy Infant Lung Development's Preterm Lung Patient Registry at Boston Children's Hospital between 2008 and 2022. The registry consists of children referred for PPRD. Inclusion criteria were birth at ≤ 32 weeks and a valid Massachusetts residential address. The Preterm Lung Patient Registry (NCT00951366) was established to identify epidemiologic and genetic factors associated with chronic lung disease among former preterm infants and those with neonatal lung disease. This study was approved by the Boston Children's Hospital Institutional Review Board (Protocol X08‐07‐0335). Informed consent to participate in the registry was obtained for each participant included in this study.

### 2.2 Exposure: Neighborhood Opportunity

Residential addresses obtained from the electronic health record were geocoded to census tracts using ArcGIS. Participants were then categorized into quartiles based on the Massachusetts state‐normalized Childhood Opportunity Index (COI) 3.0, which includes annual census data from 2012 to 2023. The COI is a validated composite measure ranging from 0 to 100 that incorporates 44 indicators across three subdomains: (1) social and economic, (2) health and environment, and (3) education. Specific indicators that comprise each subdomain are listed in Table S1. The technical methodology of constructing the COI has been described in detail elsewhere [14].

### 2.3 Outcomes: Risk Factors for Developing BPD: Birth Outcomes, Use and Duration of Respiratory Support

Demographics and NICU history were collected after enrollment in the registry. Questionnaires addressing respiratory symptoms and post‐discharge healthcare utilization were administered to caregivers in the outpatient clinic, and were similar to validated questionnaires available in the literature [20]. Subject race was included as binary caregiver‐reported demographic variable. Missing data from the child's NICU course were obtained from a chart review when necessary. Primary outcomes assessed included birth indicators: GA/BW, and the duration and type of respiratory support required in the NICU. For subjects with available pulmonary function tests (PFTs) performed between 5 and 12 years of age, spirometry data were scored based on ATS criteria [21] by the authors (JL, LH). For subjects with acceptable spirometry, the association between COI and children's *best* recorded forced expiratory volume in 1 s (FEV₁) percent predicted, forced vital capacity (FVC) percent predicted, and FEV₁/FVC ratio was assessed. We also evaluated caregiver‐reported chronic respiratory symptoms and hospital admissions, the latter serving as an indicator of excess healthcare utilization.

### 2.4 Statistical Analysis

All statistical analyses were conducted using Stata 18 (StataCorp, College Station, Texas, USA) [22]. Demographic and clinical variables were summarized and compared across COI quartiles using regression analyses, with Q1 (representing lowest opportunity) serving as the reference group. *p*‐values < 0.05 were considered statistically significant. As a preplanned secondary analysis, we examined associations between subject race and neonatal respiratory outcomes, as well as how the difference in outcomes between races varied within COI quartiles. Race was coded as a binary variable (White and non‐White) in adjusted models. To assess attenuation, we compared the regression coefficients for race before and after adding COI to the models. All figures were produced in R Statistical Software v2025.50.1 (R Core Team 2025).

## 3 Results

Residential, demographic, and clinical data were available for 845 children (Table 1). The study population was 44% female, 38% self‐identified as non‐White, and 14% as Hispanic. The mean gestational age was 26.6 ± 2.22 weeks, and the mean birthweight was 904 ± 306 g. Every subject in the cohort weighed < 2500 g and classified as being low‐birth weight (LBW), and the majority (56%) weighed < 1000 g, meeting criteria for extremely low birth weight, (ELBW). Approximately two‐thirds (66%) of the cohort required some form of respiratory support at 36 weeks, and 8% remained on MV at that time, meeting criteria for grade 3 BPD. On average, subjects spent 94.6 ± 41.7 days on respiratory support in the NICU; 27.8 ± 38.2 on MV, 34.6 ± 23.4 on CPAP, and 32.4 ± 26.1 on low‐flow oxygen (LFO_2_). Demographics are summarized in Table 1 and cohort averages of each outcome, distributed by COI quartile, are detailed In Table 2.

**Table 1:** Cohort Demographics.

Demographics (*n* = 845)	
Sex, *n* (%)	
Female	377 (44)
Male	468 (55)
Race, *n* (%)	
White	519 (61)
Non‐White	326 (39)
Ethnicity, *n* (%)	
Non‐Hispanic	669 (86%)
Hispanic	108 (14%)
Gestational Age in weeks, mean (SD)	26.6 (2.22)
Birthweight in grams, mean (SD)	904 (306)
Maternal age, mean (SD)	31.8 (5.9)
Multiple gestations, *n* (%)	309 (38%)
Maternal steroids *n* (%)	536 (82%)
**NICU course,** *n* = 731	
Days on a ventilator, mean (SD)	27.7 (37.8)
Respiratory support 36 weeks CGA, *n* (%)	
Ventilator	8%
CPAP	31%
Low Flow Oxygen	30%
Room Air	31%
Duration (days) of total respiratory support, mean (SD)	90.5 (40.7)
Ventilator	27.8 (38.2)
CPAP	34.6 (23.4)
Low Flow Oxygen	32.4 (26.1)
Received post‐natal steroids *n* (%)	205 (30%)
Received chlorothiazide (% yes)	32%
Received furosemide (% yes)	23%
**Respiratory Symptoms** (age 0–3), *n* = 398	
Wheeze (% yes)	43%
Cough (% yes)	70%
Snore (%yes)	39%
**Healthcare Utilization for Respiratory Illness** (age 0–3), *n* = 401	
Sick Visit	43%
Emergency Department Visits	32%
Hospital Admissions	22%
**Spirometry (*n*** = **83 with data)**, age 5–12, *n* = 83	
FEV1% predicted, mean (SD)	90.4 (17.0)
FVC % predicted, mean (SD)	99.6 (17.3)
FEV1/FVC, mean (SD)	83.0 (8.8)

*CGA* Corrected Gestational age. Healthcare utilization for respiratory illness represents percentage of individuals with at least one event. FEV1 Forced expiratory volume in 1s. FVC forced vital capacity.

**Table 2:** Mean Statistics of Neonatal Respiratory Indicators, by COI Quartile.

Variable	Q1 (*n* = 174)	Q2 (*n* = 175)	Q3 (*n* = 231)	Q4 (*n* = 265)	*p*‐value
**Birth outcomes** *n* = 845					
Gestational age (wks)	26.0 (25.7–26.4)	26.5 (26.2–26.8)	26.6 (26.3–26.8)	27.0 (26.7–27.2)	0.004**
Birth weight (g)	846.4 (801.9–890.9)	871.5 (824.0–919.0)	910.6 (866.2–955.0)	956.8 (919.8–993.8)	0.001***
Maternal Age	28.7 (27.5–29.9)	31.9 (30.9–32.9)	32.0 (31.3–32.8)	33.7 (33.1–34.4)	< 0.001***
**NICU Respiratory Support** *n* = 731					
Vent duration (days)	33.1 (24.0–42.3)	31.4 (24.5–38.2)	28.6 (23.9–33.3)	21.4 (18.2–24.6)	0.007**
CPAP duration (days)	37.9 (33.9–41.8)	32.3 (28.7–35.9)	36.1 (32.4–39.7)	32.5 (29.6–35.4)	0.220
Low flow oxygen Duration (days)	31.0 (27.5–34.5)	34.3 (29.1–39.5)	31.6 (27.5–35.8)	32.9 (27.0–38.9)	0.883
Total respiratory Support days	97.9 (90.2–105.5)	95.0 (85.9–104.2)	97.7 (89.8–105.6)	90.9 (82.5–99.3)	0.585

Mean statistics of infants born < 32 weeks gestation, stratified by Childhood Opportunity Index (COI) quartile. Values are presented as mean (95% confidence interval). *p*‐values reflect comparisons across COI quartiles using ANOVA or nonparametric tests, as appropriate.

Slightly less than half of the cohort had available caregiver‐reported symptom data (*n* = 398) and healthcare utilization data (*n* = 401) in early life (ages 0–3). There was an average 1.7 follow‐up forms completed per patient (range 1–6). Median age at follow‐up visit was 14 months (IQR 8–22 months). Among these, 43% reported persistent wheezing and 70% reported persistent cough. Approximately one‐fifth of the cohort was admitted to the hospital for a respiratory illness at least once during follow up. Pulmonary function testing was available for ~10% of the cohort (*n* = 83), conducted in childhood (ages 5–12). The mean percent predicted FEV₁ (FEV_1_pp) and FVC (FVCpp) for children's best performance during follow‐up were 90.4 ± 17.0 and 99.6 ±  17.3, respectively, and the mean FEV₁/FVC ratio was 83.0 ± 8.8 (Table 1). Children's best PFT performance was recorded at 6.9 years of age on average.

The majority of subjects resided in urban and suburban regions of eastern Massachusetts. Census tract COI scores ranged from 2 to 100. When divided into quartiles, there was minor variation in the distribution of subjects across neighborhood opportunity levels: 174 (21%) in Q1 (lowest opportunity), 175 (21%) in Q2, 231 (27%) in Q3, and 265 (31%) in Q4 (highest opportunity). Subject race, as defined by caregiver, was unequally distributed across quartiles. Approximately one‐half of all non‐white subjects resided in Q1, while only 11% of all white subjects resided in Q1 (*p* < 0.001).

Neighborhood opportunity was significantly associated with adverse birth outcomes known to increase the risk of developing BPD [23]. Infants in the lowest quartile of opportunity (Q1) were, on average, born 110 g lighter (846 vs. 957, *p* < 0.001), 1 week earlier (26 vs. 27, *p* < 0.001), and to mothers who were 5 years younger (29 vs. 34, *p* < 0.001) compared to those in Q4 (Table 2). Notably, all three COI sub‐domains demonstrated a significant influence on the duration of MV, BW, and GA (Figure 2), with the exception of the Health and Environment domain on BW.

**Figure 1:**
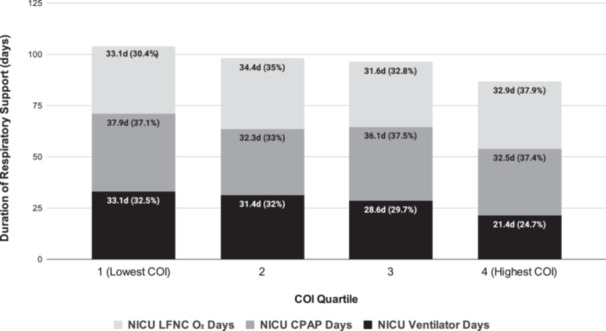
Mean Respiratory Support, by Type and COI Quartile. COI = Childhood Opportunity Index.

Significant differences were also observed in both type and duration of respiratory support. Infants in Q1 spent ~12 additional days on MV (*p* = 0.004), 5 additional days on CPAP (0.030), and 10.5 additional days on all forms of NICU respiratory support (*p* = 0.057), relative to Q4 (Table 3). Of total days on respiratory support, the proportion of invasive MV duration was greatest in Q1, and smallest in Q4 (Figure 1). There were no significant differences observed between lung function (FEV₁, FVC), healthcare utilization for respiratory illness (sick visits, emergency department visits, hospital admissions), or caregiver‐reported respiratory symptoms (wheeze, cough) across COI quartiles (Table S2).

**Table 3:** Change in birth/respiratory outcomes by COI quartile (Reference = Q1).

COI	Birth weight (g)	Gestational age (wks)	Maternal age (yrs)	Vent duration (days)	CPAP duration (days)	Low flow oxygen (days)
**Q2** (*n* = 175)	+ 25.1 (*p* = 0.458)	+ 0.47* (*p* = 0.045)	+ 3.19* (*p* = < 0.001)	−1.76 (*p* = 0.698)	−5.57 (*p* = 0.045)	+ 3.32 (*p* = 0.409)
**Q3** (*n* = 231)	+ 64.23* (*p* = 0.044)	+ 0.52* (*p* = 0.019)	+ 3.28* (*p* = < 0.001)	−4.55 (*p* = 0.281)	−1.8 (*p* = 0.478)	+ 0.62 (*p* = 0.869)
**Q4** (*n* = 265) (Highest Opportunity)	+ 110.39* (*p* = < 0.001)	+ 0.92* (*p* = < 0.001)	+ 5.01* (*p* = < 0.001)	−11.72* (*p* = 0.004)	−5.38* (*p* = 0.030)	+ 1.94 (*p* = 0.593)

Change in birth and respiratory outcomes by Childhood Opportunity Index (COI) quartile, using Quartile 1 (Q1, *n* = 174) as the reference group. Positive values indicate higher outcome values compared to Q1. Statistically significant differences (*p* < 0.05) are marked with asterisks.

Duration of MV, but not CPAP or low flow oxygen, was significantly associated with reduced FEV₁ and FVC scores in childhood (Table S3). For every additional 10 days of MV, our model predicted a 2.1% decrease in FEV₁pp (*p* = 0.001) and a 2.1% decrease in FVCpp (*p* = 0.007) (Table S3). No significant associations were observed between birth outcomes and spirometry results.

Infants admitted to the hospital during childhood spent an additional 41 days on any form of respiratory support (112 vs. 81, *p* = 0.003) and an average of 20 additional days on MV (20 vs. 40, *p* < 0.001) during their NICU course compared to those that were not. No significant differences were observed with BW, GA, or non‐invasive forms of respiratory support and hospital admissions. No associations were observed between any of the primary risk factors or secondary outcomes and caregiver‐reported symptoms.

In our preplanned secondary analysis by race, non‐White subjects were born approximately 98 g lighter (844 vs. 942, *p* < 0.001), 0.7 weeks earlier (27.1 vs. 26.8, *p* < 0.001) and to mothers 3 years younger (30 vs. 33 *p* < 0.001) when compared to White subjects. After adjusting for COI in multivariable models, subject race remained a significant predictor of birth outcomes (BW and GA). Racial disparities in birth outcomes within COI quartiles became more pronounced at higher levels of opportunity (Table 4). The impact of COI on longer‐term respiratory outcomes (lower FEV_1_pp, FVCpp, reported symptoms, and healthcare utilization) was assessed, but did not reach statistical significance (Table S2).

**Table 4:** Mean BW and GA by COI and Race (*n* = 845).

COI	Race	*n*	Birth Weight (g)	*p*‐value (BW)	Gestational Age (wks)	*p*‐value (GA)
Q1	Non‐White	138	831.6 (786.3, 876.8)	0.274	25.88 (25.55, 26.22)	0.116
(Lowest Opportunity)	White	36	900.6 (789.5, 1011.7)		26.61 (25.79, 27.43)	
Q2	Non‐White	73	875.9 (802.5, 949.3)	0.875	26.48 (25.93, 27.03)	0.887
	White	102	868.2 (810.9, 925.5)		26.53 (26.11, 26.95)	
Q3	Non‐White	59	807.6 (742.1, 873.2)	0.002*	26.34 (25.79, 26.89)	0.382
	White	172	947.0 (896.4, 997.5)		26.63 (26.29, 26.97)	
Q4	Non‐White	56	870.5 (806.4, 934.6)	0.009*	26.18 (25.66, 26.70)	0.002*
(Highest Opportunity)	White	209	979.1 (937.9, 1020.3)		27.17 (26.87, 27.47)	

Values are presented as mean (95% confidence interval) stratified by Childhood Opportunity Index (COI) quartile and race group. *indicates statistical significance (*p* < 0.05).

## 4 Discussion

Overall, in this single‐center retrospective cohort of infants with PPRD followed at an urban tertiary care center, lower COI was associated with key risk factors for developing BPD, including adverse birth outcomes and increased duration of invasive MV in the NICU. COI was not directly associated with longitudinal measures of respiratory outcomes but may have a negative modifying effect on them through its strong association with duration of invasive respiratory support (MV). Identifying and addressing modifiable risk factors is essential for primary prevention of BPD and may support optimal lung development across the lifespan [24].

The associations between low COI and adverse birth outcomes among infants who went on to develop PPRD observed in this investigation may reflect the compounded effects of chronic stress, limited access to high‐quality prenatal care, [25] poor nutrition, environmental exposures, and inadequate housing, factors disproportionately concentrated in under‐resourced neighborhoods. Environmental exposures, including air pollution and lead, may disrupt placental function and fetal development through inflammatory and oxidative stress pathways [26]. Emerging evidence has begun to elucidate potential mechanisms [27]. One population‐based study found that neighborhood adversity was independently associated with gestational epigenetic age deceleration, suggesting that structural disadvantage may influence fetal biology at the molecular level [28]. Further investigation is warranted to disentangle the relative contributions of individual COI domains to the observed birth outcomes and to identify modifiable upstream targets for intervention.

The significant difference in BW and GA across COI quartiles may appear modest at the individual level, but their implications at the population scale are substantial. For example, applying the observed difference in BW to the approximately 3.6 million US annual live births [29], a downward shift of 110 grams could result in roughly 72,000 additional infants born at LBW each year, representing a significant rise in vulnerable newborns who face elevated risks for neonatal morbidity, mortality, and long‐term health challenges [30]. However, since this study leveraged a convenience cohort, its findings may not be representative at the population level.

The difference in the distribution of total days of respiratory support in the NICU across COI quartiles was driven primarily by the most invasive form, MV (Figure 1). For example, MV accounted for an average of 33% of total respiratory support days among infants in Q1 (lowest opportunity), compared to only 25% in Q4 (highest opportunity). Longer duration of MV among infants with low COI may be a reflection the disparities in birth outcomes observed in this cohort given that low BW and early GA are strong predictors for prolonged invasive respiratory support in the NICU.

**Figure 2:**
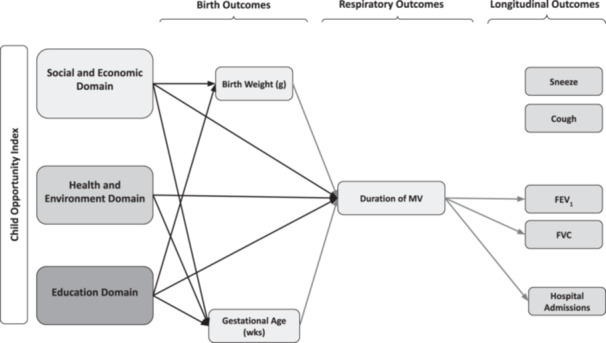
Summary of Findings. All arrows indicate significant relationship (*p* < 0.05) between IRQ increase in the respective childhood opportunity index subdomain, state normed.

In general, duration of MV in early life has significant implications for optimizing alveolar development, long‐term lung function, neurological outcomes, healthcare utilization, and subsequent medical costs. Importantly, duration of MV was significantly associated with lower FEV₁ and FVC scores in this cohort (Table S3). By mitigating exposure to prolonged invasive respiratory support, it may be possible to improve long‐term lung function and modify the downstream impact of early‐life respiratory interventions on spirometry outcomes (Figure 2). Optimizing respiratory management by addressing modifiable risk factors may also have significant implications for healthcare utilization. For example, in a subset of 201 infants in our cohort, those that were admitted to the hospital for respiratory illness in childhood, spent 20 additional days on the ventilator during their NICU course compared to those that were never admitted (*p* = 0.001). However, it remains unclear if MV is the direct risk factor for developing BPD and adverse lung development, as opposed to a proxy of respiratory severity, which would be associated with worse functional outcomes.

Interestingly, we did not observe an association between birth outcomes (BW, GA) and spirometry measures, which contrast with findings from prior studies. This discrepancy is likely attributable to the selective inclusion characteristics of our cohort, which was age restricted to infants born at ≤ 32 weeks, all of whom were born LBW.

No associations were observed between caregiver‐reported symptoms and any of the primary or secondary exposures assessed. Aside from selective limitations noted above, this null finding may reflect reporting bias, as caregivers could underestimate or underreport symptoms due to recall limitations, normalization, reluctance to raise concerns, or social desirability bias—which is differentially influenced by social, ethnic, and cultural identities [31, 32]. Such underestimation may have differentially affected quartiles of COI and biased the results toward the null, potentially obscuring true disparities in symptom burden across social opportunity groups.

Race was independently associated with birth outcomes, even after adjusting for COI. Notably, racial disparities in birth outcomes within COI quartiles were more pronounced in higher‐opportunity settings. Across all quartiles, non‐White infants were born at lower birthweights and earlier gestational ages compared to their counterparts, with these differences reaching statistical significance in the higher COI quartiles. Taken together, these findings suggest that place‐based strategies focused solely on improving neighborhood environments may be insufficient to eliminate persistent inequities in early‐life outcomes.

The interplay between individual demographics (i.e., race) and community resources on shaping neonatal health has also been supported by previous findings. In a US‐based birth cohort study, socioeconomic status (SES) was associated with increased BW and reduced infant mortality for White birthing people but had no impact on non‐White birthing people. Among preterm infants diagnosed with BPD, social vulnerability index (SVI) mediated approximately 1/3 of the population's observed Black‐White disparities in ED rates in the first year of discharge [18], demonstrating the necessity of addressing both interpersonal and structural determinants of perinatal and preterm health [33].

Evidence from other population‐based cohorts in US‐based tertiary care centers supports our primary findings. In a single‐center prospective study following infants born in the mid‐west, lower COI scores were associated with increased risks of adverse neonatal outcomes, including preterm birth, low birth weight, and NICU admissions, even after adjusting for individual‐level socioeconomic factors [34]. Among preterm infants diagnosed with BPD, post‐discharge respiratory health outcomes have been associated with increased social vulnerability [18] and elevated exposure to air pollution, using data from the CDC Environmental Justice Index (EJI) [35]. Previous studies have demonstrated relationships between COI and factors already known to influence healthy lung development in infants born preterm with BPD, including symptom burden in sleep disordered breathing [36], incidence of asthma [37], childhood obesity [38], ED visits, and use of public insurance [39].

Our study has important strengths. We were able to link individual addresses with a state‐normalized indicator of childhood opportunity to estimate geographically precise estimates of neighborhood opportunity and account for localized disparities. We were also able to obtain a large sample size and had direct access to medical records, which enabled chart review when data were missing.

Our investigation is not without limitations. This is a single‐center study of infants enrolled in a convenience cohort at a tertiary care center that provides specialized pulmonary follow‐up. Therefore, these results may not be generalized to all pediatric populations. Additionally, our data extraction retrieved only one address per patient from the electronic health record in bulk at the time of data download, limiting our ability to account for residential relocation during childhood and potentially introducing exposure misclassification. Prior research has shown that economic and racial disparities influence patterns of relocation after birth [40]. However, residential mobility tends to occur within similar neighborhood environments, such that a change in COI classification would require relocation to a census tract assigned to a different quartile. Furthermore, the use of census tract–level opportunity data may obscure individual‐level disparities, For example, a child may reside in a census tract classified in the highest quartile of opportunity, which in our model is associated with reduced risk factors for the development of BPD yet still face significant individual‐level risks such as exposure to environmental tobacco smoke which is known to impair lung development, alter immune responses to viral infections, and increase the prevalence of wheezing during childhood when present in either the prenatal or postnatal period [41, 42]. Race was also coded as a binary variable, which masks within‐group heterogeneity and oversimplifies identities. While our study evaluated duration of respiratory support in the NICU, the study did not use a formal respiratory support weaning protocol, and it is likely that individual practice variation exists. That being said, the majority of children in the study were born at a small group of NICUs in a localized area with similar practice patterns. Finally, follow‐up data on respiratory symptoms, healthcare utilization, and PFTs were only available for a subset of our original cohort, as longitudinal pulmonary care was based on clinical indication rather than through a standardized research protocol. This introduced selection bias, as higher‐risk infants were more likely to receive follow‐up, selectively enriching the sample with “sicker” individuals. Additionally, systemic social determinants, such as limited access to transportation, language barriers, and employment constraints, may have impacted families' ability to attend follow‐up appointments, potentially contributing to differential inclusion of children in the follow‐up cohort.

In the future, we aim to investigate personal measures of inequality at the individual level, rather than relying solely on proxy estimates at the census tract level—an especially important distinction in urban settings with high levels of social inequality. Leveraging data from extended follow‐up in the outpatient setting could facilitate exploration of the mechanisms by which social and environmental inequities influence neonatal care. Finally, we advocate for translational research to inform neighborhood‐level interventions and community‐based policy, which hold the potential to directly improve birth outcomes and respiratory management in the NICU and indirectly support long‐term lung function throughout childhood.

## 5 Conclusion

In this retrospective study of former very preterm infants followed for respiratory disease in the outpatient clinic, lower childhood opportunity was associated with risk factors known to influence the development BPD—including adverse birth outcomes and prolonged invasive respiratory support. Though not directly linked, indicators of long‐term pulmonary outcomes including spirometry and hospital admissions appear to be mediated by COI. These findings suggest that socio‐environmental impacts on respiratory outcomes are not limited to exposures after neonatal discharge but begin well before birth. Preventative policies should prioritize neighborhood‐level interventions with concurrent strategies that address racial equity during the prenatal period to reduce the incidence of BPD, promote optimal lung development, and advance child health.

## Author Contributions

Ella Whitman was responsible for conceptualization, data curation, formal analysis, investigation, methodology, visualization, writing (original draft and review/editing). Marissa Hauptman was responsible for methodology, supervision, and writing (review/editing). Lystra Hayden was responsible for conceptualization, data curation, resources, and writing (review/editing). Jonathan Levin was responsible for conceptualization, data curation, investigation, methodology, project administration, supervision, writing (review/editing).

## Ethics Statement

This study was approved by the Boston Children's Hospital Institutional Review Board (IRB).

## Conflicts of Interest

The authors declare no conflicts of interest.

## Supporting information


**Table S1:** COI Indicators, Grouped by Domains. **Table S2:** Mean Statistics for Longer‐Term Outcomes, by COI Quartile. **Table S3:** Association between BPD Risk Factors and Spirometry in Childhood (age 5‐12), n = 83.

## Data Availability

The data that support the findings of this study are available from the corresponding author upon reasonable request.
