# Supplementary material for: Neighborhood Opportunity and Early Life Indicators of Respiratory Health in Children Born Very Preterm
Source: Pediatr Pulmonol. 2026 Feb 9;61(2):e71501. doi: 10.1002/ppul.71501 (PMC12887135; doi:10.1002/ppul.71501)
Supplement: Supplementary file 1 — Table S1: COI Indicators, Grouped by Domains. Table S2: Mean Statistics for Longer‐Term Outcomes, by COI Quartile. Table S3: Association between BPD Risk Factors and Spirometry in Childhood (age 5‐12), n = 83. [file PPUL-61-0-s001.docx]

**Supplemental Table S1.** COI Indicators, Grouped by Domains

| **Domain** | **Subdomain** | **Indicators** |
| --- | --- | --- |
| **Social & Economic** | Economic Indicators | - Poverty rate  -Unemployment rate  - Median household income |
|  | Social Indicators | - Single-parent households  - Linguistic isolation  - Racial/ethnic diversity |
|  | Access to Services | - Availability of public transportation  - Access to healthy food options  - Proximity to libraries and community centers |
| **Health & Environment** | Access to Health Care | - Proximity to pediatricians  - Availability of health insurance  - Access to mental health services |
|  | Environmental Quality | - Air quality index  - Exposure to pollutants  - Access to green spaces |
|  | Housing & Neighborhood Conditions | - Housing vacancy rates  - Overcrowded housing  -Neighborhood safety |
| **Education** | Early Childhood Education | - Enrollment in early education programs  - Availability of early education centers |
|  | Elementary Education | - Third-grade reading proficiency  - Third-grade math proficiency  - Student-teacher ratio |
|  | Secondary & Postsecondary Education | - High school graduation rates  - College enrollment rates  - Access to advanced coursework |

Adapted from Noelke, C., McArdle, N., DeVoe, B., Leonardos, M., Lu, Y., Ressler, R.W., & Acevedo-Garcia, D. (2024). Child Opportunity Index 3.0 Technical Documentation. diversitydatakids.org, Brandeis University. Retrieved from [diversitydatakids.org/research-library/coi-30-technical-documentation](http://diversitydatakids.org/research-library/coi-30-technical-documentation).

**Table S2.** Mean Statistics for Longer-Term Outcomes, by COI Quartile

| **Variable** | **Q1** | **Q2** | **Q3** | **Q4** | **p-value** |
| --- | --- | --- | --- | --- | --- |
| **Respiratory Symptoms** (age 0-3), n = 398 | | | | | |
| **Wheeze**  **n, (%)** | 52 (53.1%) | 39 (37.1%) | 46 (46.0%) | 35 (36.8%) | 0.062 |
| **Cough**  **n, (%)** | 83 (38.3%) | 86 (39.3%) | 85 (42.5%) | 75 (35.9%) | 0.585 |
| **Healthcare Utilization for Respiratory Illness** (age 0-3), n = 401 | | | | | |
| **Sick Visits (≥1)**  **n, (%)** | 49 (49.5%) | 46 (44.2%) | 41 (40.2%) | 36 (37.5%) | 0.350 |
| **ED Visits (≥1)**  **n, (%)** | 34 (35.1%) | 35 (34.0%) | 35 (34.7%) | 24 (25.3%) | 0.411 |
| **Hospital Admissions (≥1)**  **n, (%)** | 13 (26.0%) | 11 (20.0%) | 14 (27.5%) | 6 (13.3%) | 0.326 |
| **Spirometry** (age 5-12), n = 83 | | | | | |
| **FEV_1_** | 90.01 (79.69–100.33) | 88.56 (80.59–96.53) | 92.93 (85.27–100.59) | 89.66 (84.24–95.09) | 0.857 |
| **FVC** | 92.89 (81.03–104.75) | 100.54 (91.74–109.33) | 103.19 (91.83–114.54) | 100.33 (94.27–106.38) | 0.515 |
| **FEV_1_/FVC** | 84.57 (79.26–89.88) | 82.39 (79.03–85.75) | 82.74 (78.44–87.04) | 82.71 (77.91–87.52) | 0.935 |

Values for **respiratory symptoms and healthcare utilization for respiratory illness** represent the number of participants with the outcome (percentage within quartile). p-values were calculated using Pearson’s chi-square test to assess differences across COI quartiles. COI quartiles are based on the distribution within the study population, where Q1 represents the lowest opportunity and Q4 the highest. Values for **spirometry** outcomes were calculated using one-way analysis of variance (ANOVA) to test for differences across quartiles.

**Supplemental Table S3:** Association between BPD Risk Factors and Spirometry in Childhood (age 5-12), n = 83

| **Predictor** | **FEV₁ (% predicted) β (95% CI)** | **p-value** | **FVC (% predicted) β (95% CI)** | **p-value** |
| --- | --- | --- | --- | --- |
| ***Birth Outcomes*** | | | | |
| **Birthweight (g)** | 0.00 (-0.01, 0.02) | 0.521 | 0.01 (-0.01, 0.03) | 0.431 |
| **Gestational age (week)** | -0.06 (-1.93, 1.18) | 0.948 | -0.51 (-2.76, 1.75) | 0.655 |
| ***Respiratory Support Type*** | | | | |
| **Total NICU Respiratory Support (days)** | -0.01 (-0.15, 0.13) | 0.876 | -0.04 (-0.21, 0.14) | 0.658 |
| **NICU Ventilation (days)** | -0.21 (-0.33, -0.09) | 0.001 * | -0.21 (-0.36, -0.06) | 0.007 * |
| **NICU CPAP (days)** | -0.03 (-0.20, 0.15) | 0.770 | -0.01 (-0.25, 0.22) | 0.900 |
| **NICU LFNCO₂ (days)** | 0.07 (-0.11, 0.25) | 0.418 | 0.07 (-0.16, 0.29) | 0.53 |

*indicates statistical significance (*p*<0.05)
